# Melt Processible Biodegradable Blends of Polyethylene Glycol Plasticized Cellulose Diacetate with Polylactic Acid and Polybutylene Adipate-Co-Terephthalate

**DOI:** 10.1007/s10924-023-02925-8

**Published:** 2023-05-27

**Authors:** Bethuel M. Tselana, Sudhakar Muniyasamy, Vincent O. Ojijo, Washington Mhike

**Affiliations:** 1 Polymer Technology Division, Department of Chemical, Metallurgical and Materials Engineering, Tshwane University of Technology, Pretoria, 0183 South Africa; 2 Centre for Nanostructures and Advanced Materials, DSI-CSIR Nanotechnology Innovation Centre, Council for Scientific and Industrial Research, Pretoria, 0184 South Africa

**Keywords:** Cellulose diacetate, Polyetheyelene glycol, Polylactic acid, Polybutylene adipate-co-terephthalate, Biodegradation

## Abstract

Enhancing the melt processability of cellulose is key to broadening its applications. This is done via derivatization of cellulose, and subsequent plasticization and/or blending with other biopolymers, such as polylactic acid (PLA) and polybutylene adipate terephthalate (PBAT). However, derivatization of cellulose tends to reduce its biodegradability. Moreover, traditional plasticizers are non-biodegradable. In this study, we report the influence of polyethylene glycol (PEG) plasticizer on the melt processibility and biodegradability of cellulose diacetate (CD) and its blends with PLA and PBAT. CD was first plasticized with PEG (PEG-200) at 35 wt%, and then blended with PLA and PBAT using a twin-screw extruder. Blends of the PEG plasticized CD with PLA at 40 wt% and with PBAT at 60 wt% were studied in detail. Dynamic mechanical analysis (DMA) showed that PEG reduced the glass transition of the CD from ca. 220 °C to less than 100 °C, indicating effective plasticization. Scanning electron microscopy revealed that the CD/PEG-PBAT blend had a smoother morphology implying some miscibility. The CD/PEG-PBAT blend at 60 wt% PBAT had an elongation-to-break of 734%, whereas the CD/PEG-PLA blend had a tensile strength of 20.6 MPa, comparable to that of the PEG plasticized CD. After a 108-day incubation period under simulated aerobic composting, the CD/PEG-PBAT blend at 60 wt% PBAT exhibited a biodegradation of 41%, whereas that of the CD/PEG-PLA at 40 wt% PLA was 107%. This study showed that melt processible, biodegradable CD blends can be synthesized through plasticization with PEG and blending with PBAT or PLA.

## Introduction

It is estimated that by the year 2050, 26 billion tonnes of synthetic plastics would have been generated [1]. This continued growth in the use of petrochemical-derived synthetic plastics poses numerous challenges. The raw materials for conventional synthetic plastics, crude oil, natural gas, and coal are non-renewable. Existing reserves of these fossil fuels will be depleted at some point. Also, during the production of conventional synthetic plastics, toxic chemicals such as bisphenol A are used as antioxidants and benzene, vinyl chlorides, and phthalates are used as plasticizers [2].

Most petrochemical-derived synthetic plastics are non-biodegradable, they are not decomposed naturally by microorganisms under ambient conditions [3]. This leads to the persistence of plastics waste on the environment when they are discarded as waste after their useful life has lapsed. As a result, plastics waste is a major contributor to environmental pollution.

Approximately 150–120 million tonnes of plastics waste are discarded annually worldwide [4]. Moreover, due to the covid-19 pandemic, single use plastics in the form of personal protection equipment have contributed to the generation of more plastics waste [5]. In South Africa, plastics pollution along coastal cities such as Durban has become a significant problem [6]. South Africa is ranked 11th out of 192 countries, amongst countries with high levels of plastics waste that find their way into the oceans [7].

Microplastics, which are fragments of synthetic plastics less than five millimeters in length, can be ingested or inhaled by humans, thereby accumulating in various body organs and potentially affecting human health [7]. Burning synthetic plastics waste releases toxic gases, heavy metals and other particulates that are potentially harmful, into the atmosphere. This also contributes to greenhouse gas emissions into the atmosphere [2].

Recycling is often proposed as a solution to synthetic plastics pollution. However, in recent years less than half of synthetics plastics waste collected was recycled worldwide [8]. Plastics waste contains various additivities and is often contaminated with food and other waste materials, which makes it even more expensive to recycle [3].

Challenges emanating from the use of conventional synthetic plastics have increased the interest in bioplastics made from bio-based and biodegradable polymers obtained from renewable, natural resources. The use of bioplastics is envisaged to result in less adverse environmental effects, compared to petroleum-based plastics [9].

Cellulose is a linear homopolysaccharide consisting of β-1,4 glycosidically linked D-glucopyranose (glucose) units [10]. It is the most abundant natural, biodegradable polymer on earth. It is found in plants and is also produced by microorganisms as microbial cellulose [11]. Commercially, cellulose is extracted from cotton linters and wood pulp [12].

Polymer melt processing is widely used in the manufacture of polymeric components due to its cost effectiveness and viability [13]. Conventional melt processing of cellulose is difficult due to its relatively high glass transition temperature; it tends to degrade before it softens or melts. This is due to extensive intramolecular and intermolecular hydrogen bonds in the cellulose structure [14]. Various strategies have been utilized to fabricate cellulose based plastics by modifying it. However, modification of cellulose has the potential to affect its inherent biodegradability [15].

Regenerated cellulose plastics such as cellophane film and rayon fibres are obtained through dissolution of cellulose in an appropriate solvent, forming of the products, and regeneration back into cellulose [12, 16]. The dissolution of cellulose is difficult due to its extensive hydrogen bonding [16]. Dissolution of cellulose has been traditionally achieved using harsh chemicals such as CS_2_ resulting in environmental problems. However, novel cellulose solvent systems have been under development [16, 17]. Regenerated cellulose products have the advantage of being biodegradable under aerobic composting conditions [12].

As an alternative, cellulose derivatives have been prepared through substitution of the hydrogen-bond forming hydroxyl groups of cellulose with other substituents. Cellulose esters and ethers are the most important cellulose derivatives utilized in the fabrication of cellulose based plastics. Cellulose acetate is the most industrially relevant cellulose ester [12, 13].

Complete acetylation of cellulose results in primary cellulose acetate or cellulose triacetate, in which all hydroxyl groups are substituted by acetate groups. Cellulose diacetate is a secondary acetate obtained by partial reversal of the acetylation process through hydrolysis of cellulose triacetate. Acetylation of the hydroxyl groups reduces intermolecular hydrogen bonding, increases intermolecular spacing and decreases the degree of polarity. However, cellulose acetate still decomposes below its softening temperature [12]. Hence cellulose acetate requires further modification in order for it to be melt processible. To enhance the melt processibility of cellulose acetate, suitable plasticizers can be incorporated, polymer blends with compatible polymers can be synthesized, or the cellulose backbone can be modified through for example grafting [13].

The major application of cellulose acetate is the manufacture of filter tow, although it is also used to fabricate films, plastics and fibres [18]. Although cellulose triacetate can be dry-spun into fibres from solution, it requires plasticizers to enable it to be cast as film. Cellulose diacetate can also be dry-spun into fibres from solution. It is however possible to mould or cast it as films, by using appropriate plasticizers [12].

Numerous plasticizers for cellulose acetate have been studied [19]. Dimethyl phthalate, which was widely used in the late twentieth century, was mostly replaced by diethyl phthalate due to its high volatility [12]. Plasticizers can bleed out of the plasticized cellulose acetate in the long-term, resulting in their exposure to the environment and negatively affecting the properties and appearance of the cellulose acetate. Phthalate and phosphate plasticizers are known to be toxic, their wide scale use has thus decreased due to health and environmental concerns [12, 20].

Nowadays, the focus has now shifted to more eco-friendly plasticizers. For instance, Ghiya et al. [21] successfully melt compounded cellulose acetate with triethyl citrate and acetyl triethyl citrate. They observed an increase in the biodegradation rate with an increase in plasticizer content. Quintana et al. [22] plasticized cellulose acetate through solvent casting and melt processing using the eco-friendly plasticizers triacetin, tripropionin, triethyl citrate, tributyl citrate, tributyl 2-acetyl citrate and polyethylene glycol (PEG). Wang et al. [23] used PEG200 as an eco-friendly plasticizer for cellulose acetate and compared its plasticization efficiency with that of triethyl citrate, using melt extrusion. Kimura et al. [24] studied the rheological proprieties of triethyl citrate plasticized cellulose acetate, thereby confirming its melt processibility. Ionic liquids are also increasingly being studied as eco-friendly plasticizers for cellulose acetate due to their low toxicity and high thermal stability, amongst other interesting properties [25]. Bendaoud, Chalamet [26] demonstrated the feasibility of melt processing of cellulose acetate plasticized with an ionic liquid.

Structural features of the cellulose acetate in combination with environmental factors are known to influence its rate of biodegradation [20]. For instance, it is known that the biodegradation rate of the cellulose acetate decreases with an increase in the degree of substitution of the cellulose acetate [18]. However, the effect of plasticization on the biodegradation rate of cellulose acetate is yet to be extensively understood [20]. Phuong et al. [27] showed that plasticization of cellulose diacetate using diacetin, triacetin or their combination at 30 wt% enabled its melt processing and also increased its biodegradation rate.

Although PEG has been studied as a plasticizer for cellulose acetate, its effect on the biodegradation of PEG plasticized melt processible cellulose acetate has not yet been widely reported upon [23]. PEG is a linear polyether of ethylene glycol (ethane-1,2-diol) with a relative molecular weight of 200 mol/g (PEG200) and above [28]. It is considered to be non-toxic and biodegradable [23].

In a study by Buchanan et al. [29], cellulose acetate blends plasticized with PEG400 were observed to have an accelerated biodegradation rate. Nigam et al. [30] observed that PEG600 accelerated the biodegradation rate of PEG600 plasticized solution cast films.

Cellulose esters with short chains such as cellulose acetate have been reported to be highly miscible with aliphatic biodegradable polyesters [14, 31]. However, blends of cellulose esters such as cellulose diacetate with biodegrable polyesters and co-polyesters such as polylactic acid (PLA) and polybutylene adipate-co-terephthalate (PBAT) respectively, are yet to be reported on. Challenges associated with the blending of cellulose derivatives with other polymers include the need to enhance adhesion between phases and the reduction of the interfacial tension between blended polymers [32].

The present study was focused on enhancing the melt-processibility of the cellulose derivative cellulose diacetate (CD)*,* in pursuit of developing biodegradable plastics. The study considered the utility of polyetheyelene glycol with a molecular weight of 200 g/mol (PEG 200) as a biodegradable plasticizer in enhancing the melt processibility of CD, and its effect on the biodegradation rate of CD. Furthermore, the effecting of blending the plasticized CD with the biodegradable polymers PBAT and PLA on melt processibility, mechanical properties and biodegradation was investigated. In the study, the thermal, structural, and mechanical properties of the PEG plasticized CD and its blends with the biodegradable polymers were investigated and reported upon. In addition, biodegradation tests using simulated aerobic industrial composting were carried out on the PEG plasticized CD and its blends with the biodegradable polymers.

## Materials and Methods

### Materials

Cellulose diacetate (CD) in powder form (Degree of substitution 2.4, M_n_ 64,787 Da, 54.7% combined acetic acid content) was supplied by Haohang Industry (China). Polyethylene glycol (M_w_: 200 g/mol, 99%) (PEG) was supplied by Sigma Aldrich. PLA and PBAT were used as the biodegradable blending polymers. The PLA was grade Luminy L175 (MFI 3 g/10 min (at 190 °C/2.16 kg), density 1.24 g/cm^3^) obtained from Total Energies Corbion. PBAT was grade Ecoflex F Blend C1200 (MFI 2.5–4.9 g/10 min (at 190 °C/2.16 kg), density range 1.25–1.27 g/cm^3^) obtained from BASF.

## Methods

### Plasticization of Cellulose Diacetate with PEG200

The CD powder was dried in an oven at 60 °C for 24 h prior to mixing with the plasticizer. Thereafter, the PEG plasticizer was premixed with the CD powder using a 750 W heavy duty grinder-mixer (Preethi Blue Leaf Gold, model MG-1150). Batch sizes of 300 g of CD and the plasticizers were prepared. The plasticizer content was kept at 35 wt% as optimum content after several extrusion trials. The mixing time used was 30 s. The CD powder premixed with the PEG plasticizer was left to dry at room temperature conditions for 20 min after high-speed mixing, thereby producing a fine particulate powder. It was then subsequently dried at 60 °C in an oven for 12 h prior to extrusion.

The CD samples premixed with the PEG plasticizer were then subsequently extruded using a Ninjing Giant A SHJ-20 co-rotating twin screw extruder with a screw L/D ratio of 40. A temperature profile of between 120 and 160 °C from the extruder feed section to the die was used in the extrusion. A screw speed of 40–45 rpm was used. These extrusion parameters were selected after several trials to ensure that there was no obvious degradation of the cellulose diacetate, that is, there was no noticeable yellowing or burning wood smell of cellulose. The feed speed and pelletizer speed were varied between 12 and 16 and 4.5–5 rpm, respectively. The extrude was cooled in a water bath, dried using a blower, and then pelletized. The plasticized CD pellets were then stored in an airtight plastic bag, in an oven maintained at 60 °C, prior to further processing and characterization.

### Blending of PEG Plasticized CD with PLA and PBAT

Good melt-processablity with respect to extrusion was observed at a minimum PEG plasticizer content of 35 wt%, hence the 35 wt% PEG plasticized CD was selected for blending with PLA and PBAT. Compositions of the PEG plasticized CD with PLA and PBAT were premixed thoroughly in a plastic bag. Table 1 shows the formulations considered in preparing the blends. The mixtures were then kept in an oven at 50 °C for 24 h prior to extrusion. The blends were then extruded using similar processing parameters used during the plasticization of CD with PEG.

**Table 1:** Formulations of PEG plasticized CD blends with PLA and PBAT

35 wt% PEG- plasticized CD (wt%)	PBAT (wt%)	PLA (wt%)
100	–	–
80	20	–
60	40	–
40	60	–
20	80	–
80	–	20
60	–	40
40	–	60
20	–	80

### Injection Moulding of the Neat Polymers, PEG Plasticized CD and the Blends

The PEG plasticised CD, neat PLA and PBAT and their blends with the PEG plasticized CD, were injection moulded into tensile testing specimens using a TMC-30H injection moulding machine. Table 2 shows the parameters used for injection moulding.

**Table 2:** Injection moulding parameters for tensile test specimens

Parameter	Set Value
Temperature profile	180–200 °C
Back pressure	10 bar
Injection pressure	95 bar
Cooling time	60 s
Mould temperature	25–35 °C

### Characterization

#### Thermogravimetric Analysis

The thermal stability of the neat polymers, the PEG plasticized CD and its blends with the PLA and PBAT was evaluated using thermogravimetric analysis (TGA). The analysis was performed using a TGA Q5500 instrument from TA instruments at a heating rate of 10 °C /min from room temperature to 800 °C under an air atmosphere with a flow rate of 60 mL/min. Sample weight was maintained at 10 ± 0.5 mg.

#### Dynamic Mechanical Analysis

Dynamic mechanical analysis (DMA) was utilized to study the plasticization effect of the PEG on the CD. DMA was conducted on a Perkin Elmer DMA 8000. A dual cantilever mode was used at a frequency of 1 Hz, at a heating rate of 3 °C/min in the temperature range of − 100–150 °C under a nitrogen atmosphere. Specimens for DMA were prepared on a Carver hot press. Samples in pellet form were heated at 190 °C for 5 min without pressure and for 5 min under a pressure of 1 bar. The samples were allowed cool to room temperature prior to analysis. The CD powder (ca. 11 mg) was enclosed in a folded metal plate “material pocket” for analysis. Sample dimensions were 24.5 × 10.00 × 1.5 mm^3^. A single cantilever mode was used at a frequency of 1 Hz, at a heating rate of 3 °C/min in the temperature range – 100–250 °C.

#### X-Ray Diffraction Analysis

For x-ray diffraction (XRD) analysis, the CD powder was compressed into a pellet. Small flat plates cut from injection moulded samples of the neat PLA and PBAT, PEG plasticized CD and its blends with PLA and PBAT were used for the analysis. These were analysed using the X’Pert PRO PW3040/60 x-ray diffractometer from PANalytical. The operating current and voltage were 40 kV and 45 mA, respectively. Cu Kα radiation with a wavelength of 0.1540598 nm was used. The diffraction angles were from 5 to 90° at a scan rate of 5°/min.

#### Fourier-Transform Infrared Spectroscopy

A Perkin-Elmer Spectrum 400 FTIR spectrometer, fitted with an attenuated total reflectance (ATR) accessory, was used to determine the infrared spectra of CD, PBAT, PLA and the PEG plasticized CD and its blends with PBAT and PLA. The spectra were recorded in the infrared absorbance region of 550–4000 cm^−1^ with a resolution of 4 cm^−1^, using sixteen scans.

#### Scanning Electron Microscopy

The morphologies of the PEG plasticized CD and its blends with PLA and PBAT were investigated using the Jeol JSM-7500F field emission scanning electron microscope. The specimens were cryogenically fractured after immersion in liquid nitrogen, allowing the observation of the sample cross-sections. The fractured cross sections to be observed were coated with carbon by sputtering to avoid electron charge build-up during observation. The instrument was operated at an acceleration voltage of 3 kV to acquire secondary electron images.

#### Tensile Properties

Tensile properties of the neat PLA, PBAT, PEG plasticized CD and its blends with PLA and PBAT were determined on a Lloyds RX universal testing machine fitted with a 5 kN load cell. A cross head speed of 50 mm/min was used according to the ASTM D638 (2014) standard. Injection moulded dumbbell shaped specimens with a gauge length of 50 mm, thickness 3.75 mm, width 10 mm were used in the tests. An average of 6 specimens were tested.

### Biodegradation Tests

#### CO_2_ Biodegradation Tests

The ASTM D5338 standard test method was used to determine the biodegradability of test samples under industrial composting at 58 ± 2.0 °C [33]. A well aerated 3 months old organic rich compost obtained from Garden Master, Pretoria, South Africa was used for the compost biodegradation studies. The compost was passed through a sieve with a mesh of < 0.8 cm to achieve a uniform particle size for the biodegradation study. Table 3 shows the physicochemical properties of the compost. The activity of the compost was measured based on volatile solids of the compost. The compost produced 62.1 mg of carbon dioxide per gram of volatile solids over the first 10 days as per standard requirements.

**Table 3:** Physicochemical properties of the compost used in this study

Analysis	Compost
Total dry solids (%) ^1^	48
Volatile solids (%) ^2^	33
pH of compost solution	7.5
Total organic carbon content (%)^3^	15.15
Total Nitrogen (%)^3^	0.5
Carbon/Nitrogen ratio	30.3

^1^ The amount of solids obtained by taking a known volume of compost and drying at about 105 °C for 10 h; ^2^ The amount of solids obtained by subtracting the residue of a known volume of compost after incineration at about 550 °C for 30 min; ^3^ Elemental analysis.

**Fig. 1:**
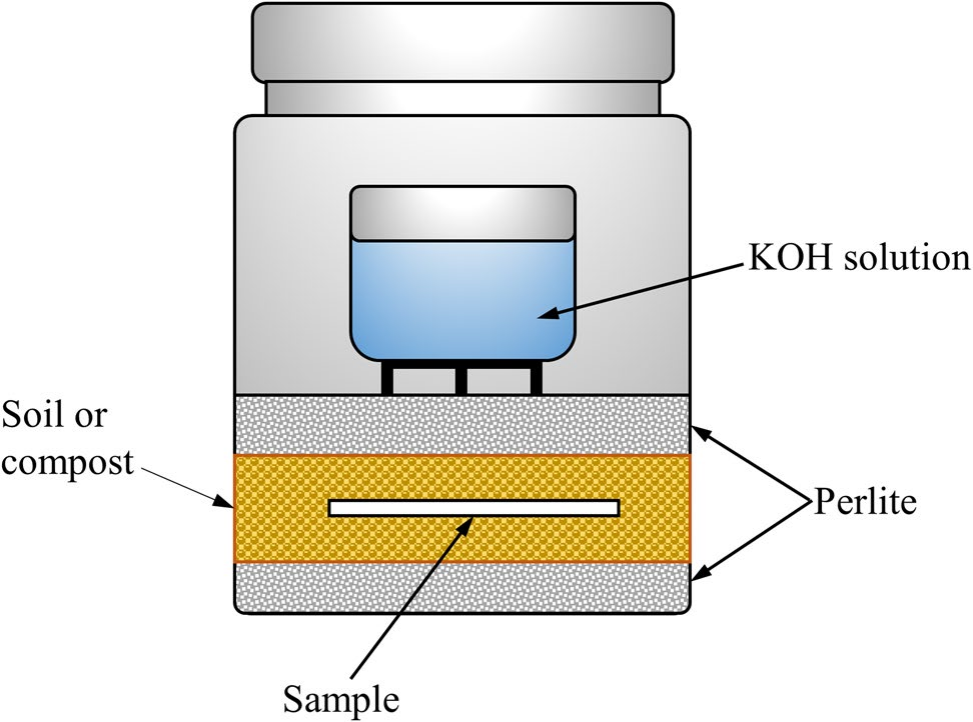
Schematic diagram of respirometric biodegradation flask system

The ultimate biodegradability (CO_2_ evolution) of test sample films was tested under composting conditions alongside with microcrystalline cellulose as a positive reference in three replicates as per the ASTM D5338 method. The biodegradability of the test materials was analyzed in biometer respirometric flasks as shown in Fig. 1 by adopting the following procedures [34].

The polymer sample particles were added to the compost mixture in a ratio of 1:6 (w/w sample to dry solids of compost). The mixture was then placed in a hermetic glass flask with a capacity of 1000 mL. The mixture was contained between a bottom layer of 20 g of perlite, wetted with 20 g of water, along the bottom of the flask, and an upper layer of 20 g of perlite, wetted with 20 g of water, covering the compost mixture to preserve moisture content. Finally, a beaker containing 40 mL of potassium hydroxide solution (0.5 N KOH) was placed on the upper layer to trap emitted carbon dioxide. A control (blank) bioreactor was also prepared (without any sample).

The CO_2_ produced during the biodegradation process was trapped in 40 mL of 1 M KOH. The CO_2_ traps were changed every 2–3 days, depending on the degradation rate of the sample material. A 10 mL aliquot of KOH from each trapping solution was titrated with 1 M HCl, using phenolphthalein indicator. The total amount of CO_2_ produced by the sample was calculated with reference to the amount of CO_2_ produced by the control flask. Throughout the test period, the compost moisture content was maintained between 50 and 55% relative humidity by adding water after every 2 weeks. This set-up was incubated at a constant temperature of 58 ± 2 °C and maintained throughout the 108 days of the experiment. The glass flasks were shaken and weighed weekly to ensure proper aeration and mixing of the bio-waste.

The total CO_2_ emitted from each reactor during the test conditions was considered as the total degradation of test sample. The biodegradation of test sample was calculated based on the total organic carbon (*C*_*t*_) present in the test samples as determined by elemental analysis.

Equation 1 below was used to calculate the theoretical CO_2_ (CO_2_(t)) in the total dry weight of plastic material.

1 $${CO}_{2 }(t)={M}_{t}\times {C}_{t}\times \frac{44}{12}$$

In Eq. 1, *M*_*t*_ is the total dry weight of plastic material added to the compost, and *C*_*t*_ is the relative weight of the total organic carbon in the dry plastic material. The percentage biodegradation of the test sample’s organic carbon mineralized as CO_2_ was calculated according to the expression in Eq. 2, where *(CO*_*2*_*)s* is the carbon dioxide from the test sample (compost + specimen), *(CO*_*2*_*)c* is the carbon dioxide from the blank compost and *(CO*_*2*_*)t* is the total theoretical amount of carbon dioxide in the test material. A biodegradation curve was obtained by plotting the percentage biodegradation versus incubation time.

2 $${\text{Biodegradation}}\left( \% \right) = \frac{{\left( {CO_{2} } \right)s - \left( {CO_{2} } \right)c}}{{\left( {CO_{2} } \right)t}} \times 100$$

#### Disintegration Biodegradation Tests

Disintegration tests monitor the primary degradation step to determine whether the material breaks into small pieces or not. The disintegration of test films was carried out in the same compost used for the CO_2_ biodegradation tests under controlled composting conditions (at 58 ± 2.0 °C). About 5 g of polymer pellets (powder for CD) were weighed into a glass-watch. About 20 mL of chloroform solvent was added to dissolve the samples. The solvent was then evaporated in a fume hood to form films. The developed film samples were then placed on top of the compost in a bioreactor. The fragmentation/disintegration of test samples at frequent time intervals were monitored for up to 70 days.

## Results and Discussions

### Mechanical Properties

Tensile tests were conducted on the neat PLA, PBAT, PEG plasticized CD and the blends of the PEG plasticized CD with PLA and PBAT synthesized through extrusion and fabricated into tensile test specimens using injection moulding, in order to screen the blends for further studies.

**Fig. 2:**
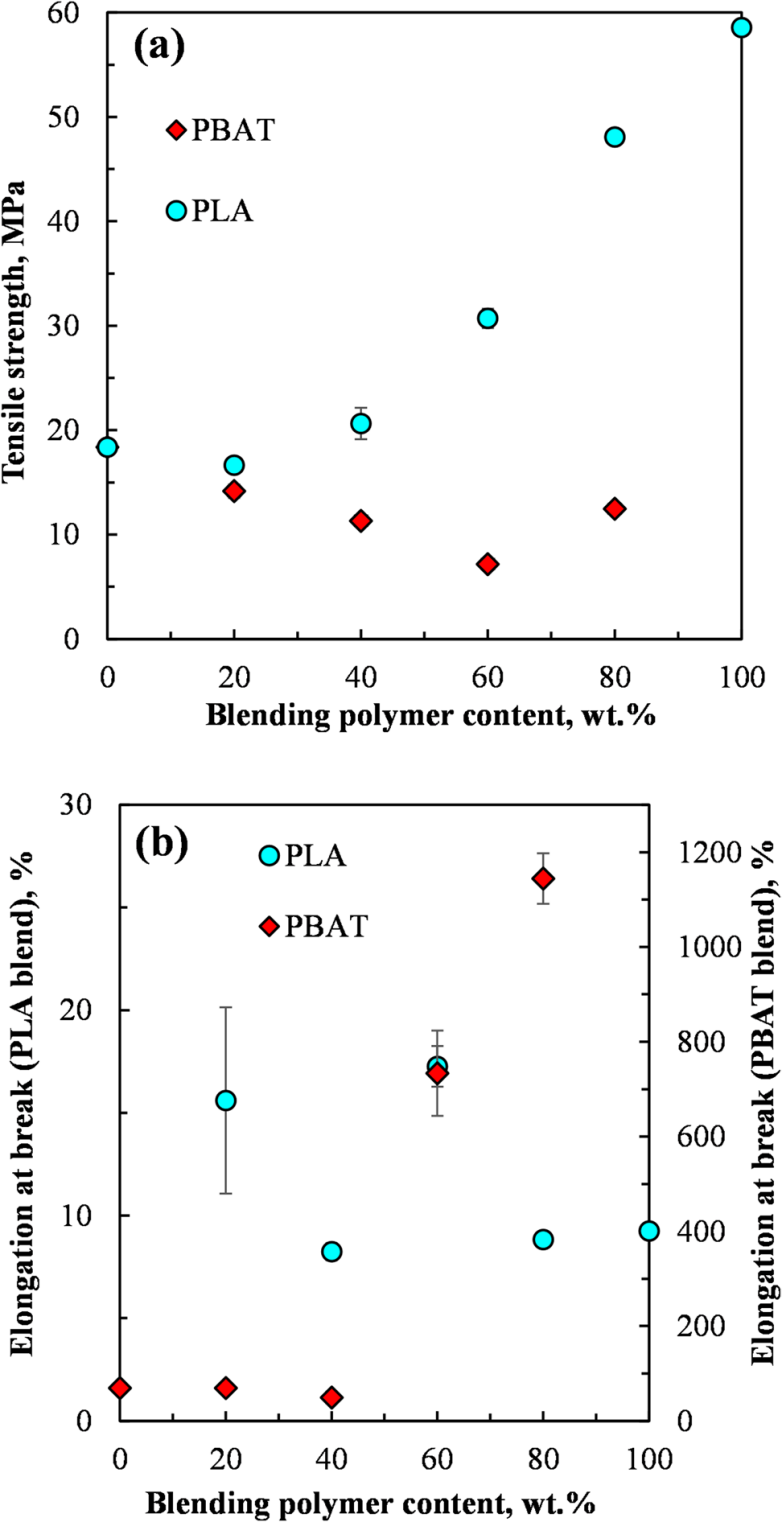
**a** Tensile strength of PEG plasticized CD blended with PBAT and PLA and **b** Elongation at break of PEG plasticized CD blended with PBAT and PLA

**Fig. 3:**
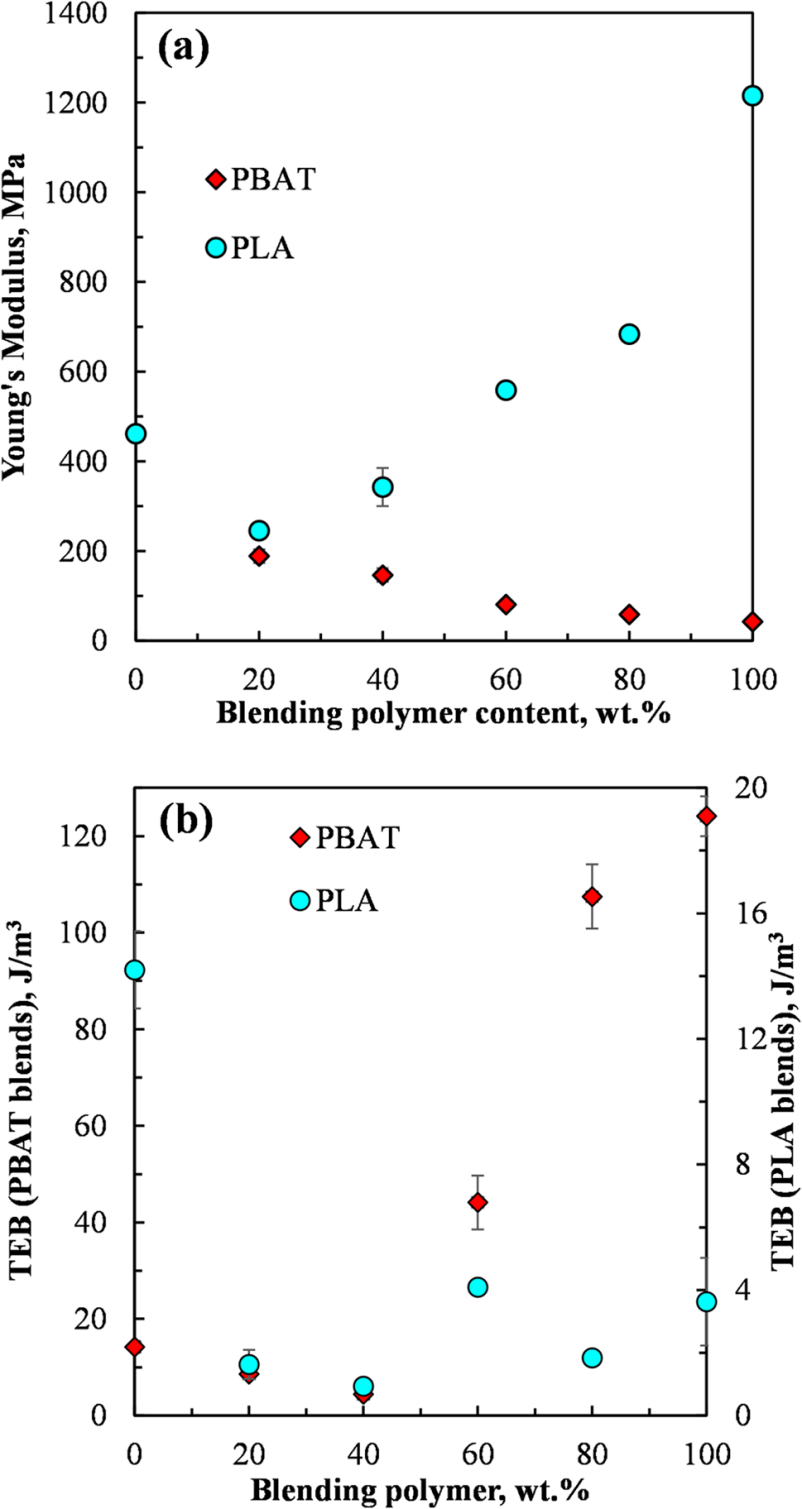
**a** Young’s modulus of PEG plasticized CD blended with PBAT and PLA and **b** Tensile energy to break (TEB) of PEG plasticized CD blended with PBAT and PLA

Figures 2a and b, 3a and b and Table 4 show the mechanical properties of the PLA, PBAT, PEG plasticized CD and the blends of the PEG plasticized CD with PLA and PBAT.

**Table 4:** Mechanical properties of PBAT, PLA, PEG plasticized CD (CD65-PEG35), PEG plasticized CD blended with 60 wt% PBAT ((CD65-PEG35)40-PBAT60) and PEG plasticized CD blended with 40 wt% PLA ((CD65-PEG35)60-PLA40)

Sample	Tensile Strength (MPa)	Young’s Modulus (MPa)	Elongation at Break (%)	Tensile Energy to Break (J/m^3^)
CD65-PEG35	18.4 ± 0.6	461 ± 17	92 ± 6	14.2 ± 1.2
PBAT	Did not break	42.4 ± 2.7	Did not break	> 124.1 ± 4.1
PLA	58.6 ± 0.6	1215.9 ± 2.9	9.2 ± 0.2	3.6 ± 1.4
(CD65-PEG35)40-PBAT60	7.2 ± 0.2	81.2 ± 6.1	733.8 ± 89	44.1 ± 5.6
(CD65-PEG35)60-PLA40	20.6 ± 1.5	342.5 ± 42.6	8.2 ± 0.4	0.9 ± 0.1

Figure 2a shows that the tensile strength of PEG plasticized CD blended with PLA increased with an increase in the PLA content. However, the tensile strength of PEG plasticized cellulose blended with PBAT decreased with an increase in the PBAT content, exhibiting a minimum at 60 wt% PBAT. Figure 2b shows that the elongation at break decreased with an increase in the blending polymer content for all the CD plasticized with 35 wt% PEG blends except for the blends with PBAT at 60 wt% and 80 wt% content PBAT.

The tensile strength of polymer blends is known to be highly sensitive to their interfacial interactions, and hence their miscibility [35]. Highly miscible polymer blends, that is, those with strong interfacial interactions, exhibit a monotonous increase or an upper bound in their tensile strengths and strains with a variation in their composition, whereas partially miscible or immiscible blends show a minimum in their tensile strengths and strains with a variation in their compositions [35, 36]. Thus, a lower bound in the variation of tensile strength with the blend composition is evidence of partially miscible or immiscibility in the blends due to weaker interfacial interactions. Based on this premise, the tensile strength data of the blends presented in Fig. 2a suggests that the CD plasticized with 35 wt% PEG blends with PLA and PBAT were either partially miscible or immiscible.Fig. 3a shows that neat PBAT had a lower Young’s modulus (42 MPa) compared to that of the PEG plasticized CD (462 MPa), whereas PLA exhibited a higher Young’s modulus of 1216 MPa. The results showed that the Young’s modulus of the PEG plasticized CD blended with PBAT decreased monotonously with an increase in the PBAT content, whereas the PEG plasticized CD blended with PLA exhibited a minimum Young’s modulus at 20 wt% PLA, which however subsequently increased with the PLA content. Previous studies have shown that the Young’s moduli of heterogenous polymer blends were dependent on the modulus of the components of the blends and their composition in the blends, and was less sensitive to the interfacial interactions [35].

Figure 3b shows that the tensile energy to break of the PEG plasticized CD blended with PBAT decreased with the PBAT content, up to 40 wt% PBAT. Further increases in the PBAT content resulted in an increase in the tensile energy to break of the blends. The tensile energy to break is a measure of the toughness of the blends. Thus Fig. 3b shows that the PEG plasticized CD was much less tough compared to PBAT. A PBAT weight content of at least 60 wt% was required to enhance the toughness of the PEG plasticized CD/ PBAT blends by more than 200%.

Figure 3b also shows that the tensile energy to break of the PLA and hence its toughness was significantly lower than that of the PEG plasticized CD. Although increasing the content of the PLA in the PEG plasticized CD blends with PLA resulted in lower tensile energy to break, values, no discernable trend was apparent with a change in the PLA content.

Two formulations, PEG plasticized CD blended with 60 wt% PBAT (CD65-PEG35)40-PBAT60)) and PEG plasticized CD blended with 40 wt% PLA ((CD65-PEG35)60-PLA40) were selected for further study based on their mechanical properties observed in Figs. 2 and 3, and Table 4. (CD65-PEG35)40-PBAT60 had a relatively high ductility, as shown by its elongation to break in Fig. 2b and Table 4. (CD65-PEG35)40-PBAT60 also exhibited an appreciable level of toughness (Fig. 3b and Table 4.) Fig. 2 a and Table 4 also shows that the tensile strength of (CD65-PEG35)60-PLA40 was comparable to that of the PEG plasticized CD. Table 4 summaries the mechanical properties of the two selected blends as well as those of the neat PLA, PBAT and the PEG plasticized CD.

### Dynamic Mechanical Analysis of Plasticized CD

Plasticizers enhance the melt processability of polymers by decreasing their glass transition temperatures and melt viscosities. DMA was used to determine the glass transition temperature (T_g_) of the unplasticized and plasticized CD, thereby evaluating the effectiveness of the biodegradable plasticizer considered for plasticizing CD in this study.

**Fig. 4:**
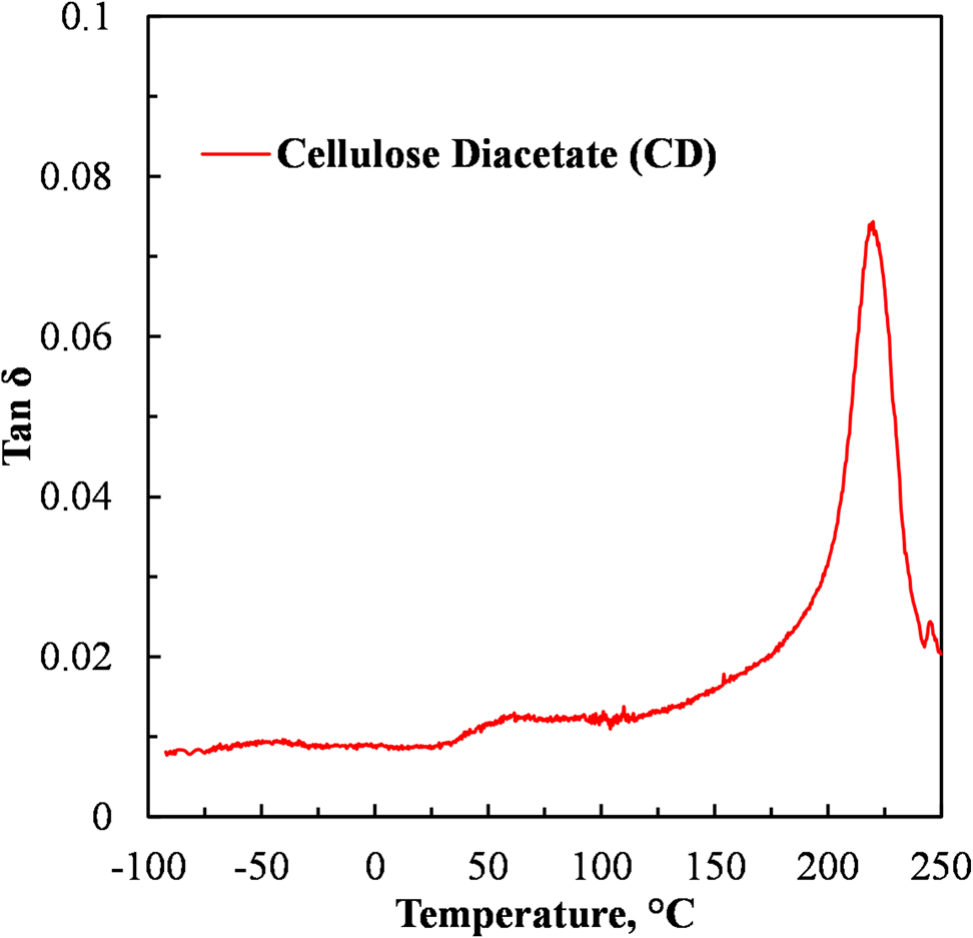
Dynamic mechanical analysis of cellulose diacetate (CD)

Figure 4 shows the tanδ against temperature curve obtained in the DMA of the neat CD. A peak on the tanδ curve indicates the T_g_ of a polymer. Cellulose and its derivatives are known to have an α- relaxation (main T_g_) and a β- relaxation region [37]. The DMA results presented in Fig. 4 show that the T_g_ of the CD used in this study was about 220 °C. This T_g_ is comparable to the T_g_ values of around 190–226 °C which were obtained in previous studies for CD with degrees of substitution of between 2.45 and 2.50 [19, 37, 38].

**Fig. 5:**
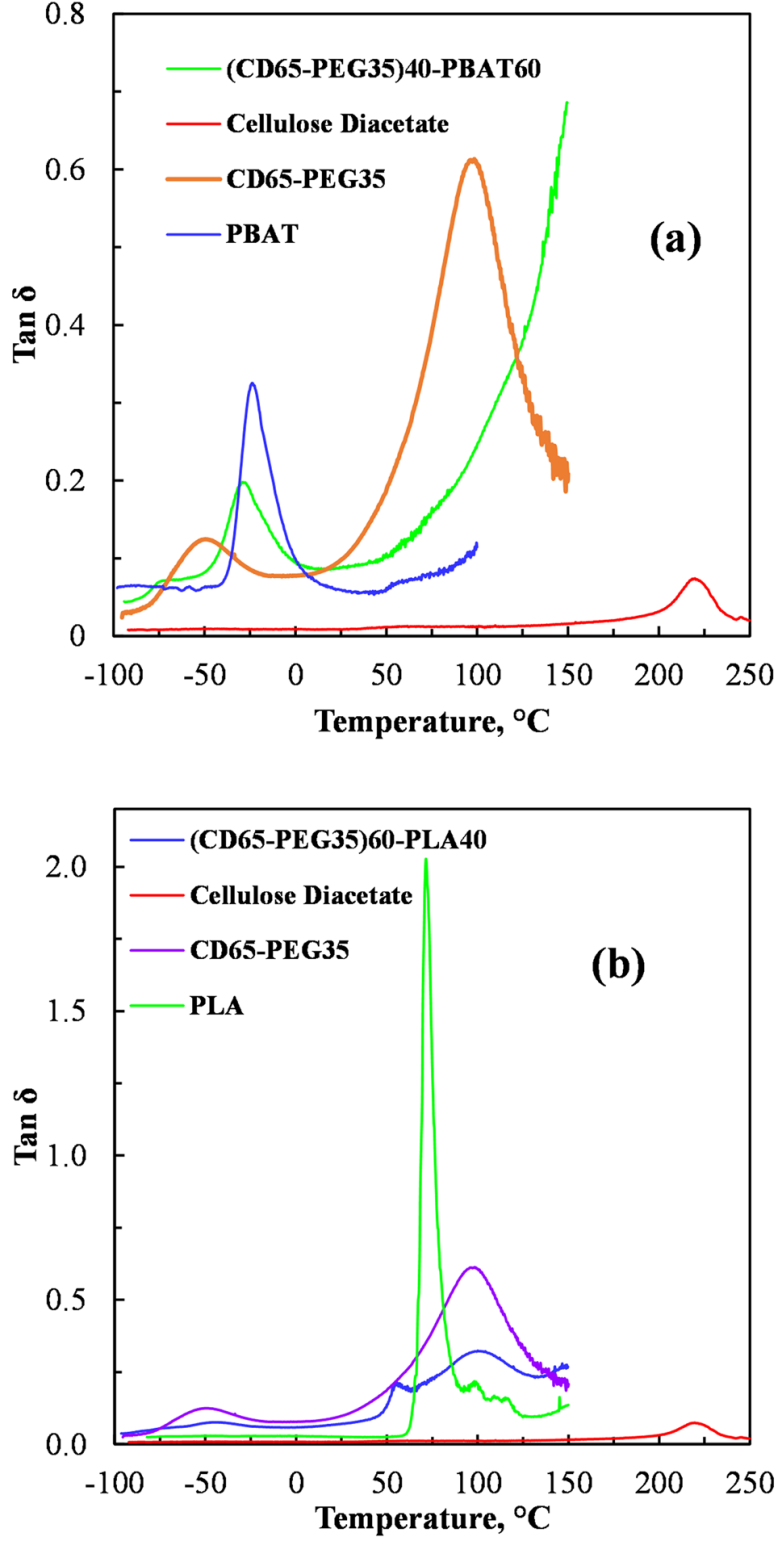
DMA tanδ curves of **a** CD, PEG plasticized CD (CD65-PEG35), PBAT and PEG plasticized CD blended with PBAT at 60 wt% ((CD65-PEG35)40-PBAT60)) and **b** CD, PEG plasticized CD (CD65-PEG35), PLA and PEG plasticized CD blended with PLA at 40 wt% ((CD65-PEG35)60-PLA40)

In Fig. 5 the DMA tan δ curves of the two selected blends superimposed on those of the neat polymers CD, PBAT, PLA and the PEG plasticized CD are presented. Figure 5a shows a prominent peak at ca. − 25 °C in the tanδ curve of the PBAT, which is identified as its T_g_. Figure 5a also shows the prominent peak at ca. 220 °C in the tanδ curve of the CD, which was earlier on identified as the T_g_ of the CD in Fig. 4. Figure 5a further shows that the T_g_ of the PEG plasticized CD was ca. 100 °C. The peak observed in the tanδ curve of the PEG plasticized CD at ca. − 50 °C is attributed to the melting transition of the PEG.

The tanδ curve of PEG plasticized CD blended with PBAT at 60 wt% in Fig. 5a shows a single prominent peak at ca. − 30 °C. This transition is very close to the T_g_ of PBAT, which was observed at ca. − 25 °C. The peak at − 30 °C was apparently an amalgamation of the melting transition of PEG and the glass transition of PBAT. It is likely that the transitions due to CD were not apparent in the tanδ curve of PEG plasticized CD blended with PBAT at 60 wt% BAT, since this blend was rich in the PBAT. The transitions due to PBAT would likely mask those due to the CD, if they occurred around the same temperature. For instance, from Fig. 5a it is observed that the PBAT started to melt at ca. 100 °C.

Miscible polymer blends exhibit a single T_g_, whereas immiscible polymer blends exhibit separate glass transition temperatures [39, 40]. The analysis on the tensile strength of PEG plasticized CD blends with PBAT suggested that the blends were either partially miscible or immiscible. A single T_g_ was not apparent in the results presented in Fig. 5a, confirming that the 35 wt% plasticised CD blend with PBAT at 60 wt% was either partially miscible or immiscible.

Figure 5b shows a prominent peak at ca. 75 °C in the tanδ curve of the PLA, which was identified as its T_g_. It also shows a prominent peak at ca. 220 °C in the tanδ curve of the PEG plasticized CD, which was earlier on identified as the T_g_ of the CD. Figure 5b also shows that the T_g_ of the PEG plasticised CD (CD65-PEG35) was ca. 100 °C.

The tanδ curve of PEG plasticized CD blended with PLA at 40 wt% in Fig. 5b shows two peaks at ca. 55 °C peak and ca. 100 °C, with the peak at ca. 100 °C rather broad. The two peaks in the tanδ curve of the blend appear to represent the glass transition temperatures of the PEG plasticized CD and the PLA. It was thus deduced that at this composition this blend was either partially miscible or incompletely immiscible. The analysis on the tensile strength of PEG plasticized CD blends with PLA, suggested that the blends were partially miscible or immiscible. Figures 5a and b show that PEG was effective in reducing the T_g_ of CD hence improving its thermal processability.

### Thermogravimetric Analysis

**Fig. 6:**
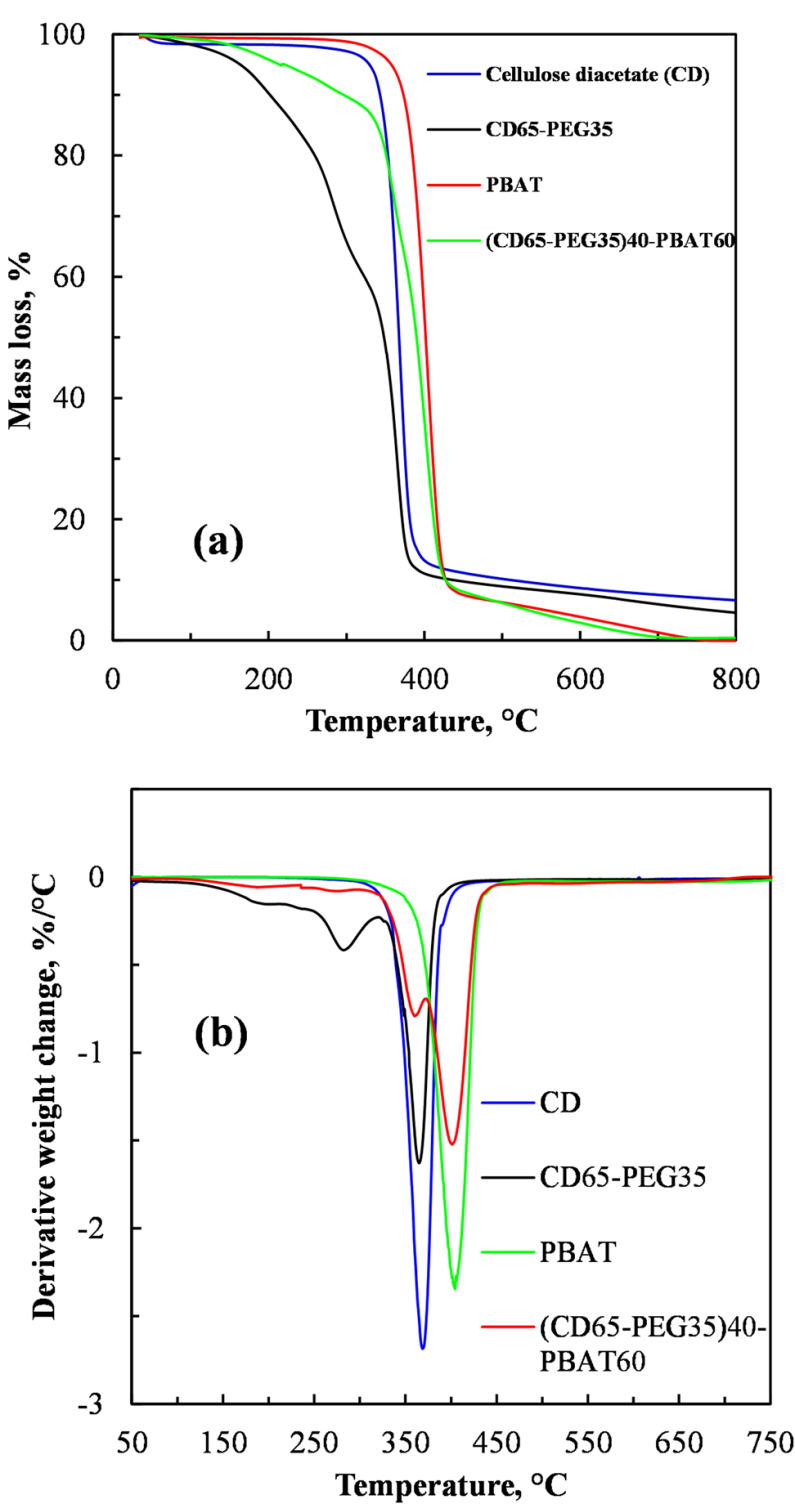
**a** TGA and **b** derivative TG of CD, PEG plasticized CD (CD65-PEG35), PBAT and PEG plasticized CD blended with PBAT at 60 wt% ((CD65-PEG35)40-PBAT60)

Figure 6 shows the thermal stability of the neat CD, the PEG plasticized CD, PBAT and the PEG plasticized CD blended with PBAT at 60 wt%, studied through TGA (Fig. 6a) and the derivative TGA (Fig. 6b). Figures 6a and b show that CD exhibited a two-step thermal decomposition mechanism. At around 100 °C there was slight mass loss due to evaporation of absorbed moisture. Its onset of thermal degradation temperature was greater than 330 °C, similar to onset degradation temperatures of CD of between 320 and 340 °C reported elsewhere [41].

Figures 6a and b show that the PEG plasticized CD exhibited a three-step decomposition mechanism. It showed a slight mass loss due to evaporation of absorbed moisture around 100 °C. Between 100 and 300 °C there was appreciable mass loss of about 30%. Compared to polymers, plasticizers are relatively low molecular weight compounds. Lower molecular weight PEGs are reported to degrade below 200 °C [42, 43]. However in the presence of oxygen, PEG-400 has been reported to start degrading at 80 °C mainly due to oxidation [42]. The bulk of the degradation (50% mass loss) of the PEG plasticized CD occurred around 320–390 °C, similar to the degradation of the neat CD. This was the thermal decomposition of the main CD molecular chains. The last step of thermal degradation of the plasticized CD involved carbonization of degraded products into ash at temperatures above 480 °C [44].

Figures 6a and b also shows that the onset temperature for thermal degradation for the 35 wt% plasticized CD blended with PBAT at 60 wt% ((CD65-PEG35)40-PBAT60) was around 150 °C, which was slightly higher than the onset temperature of the of the CD plasticized with 35 wt% PEG. However, the onset temperature for degradation of the blend was lower by almost 200 °C, compared to that of the neat CD and the neat PBAT. These observations indicated that plasticizing the CD with the polyethene glycol at 35 wt% PEG deteriorated the thermal stability of the CD. However, blending the CD plasticized with 35 wt% PEG with PBAT at 60 wt% slightly improved its thermal stability.

**Fig. 7:**
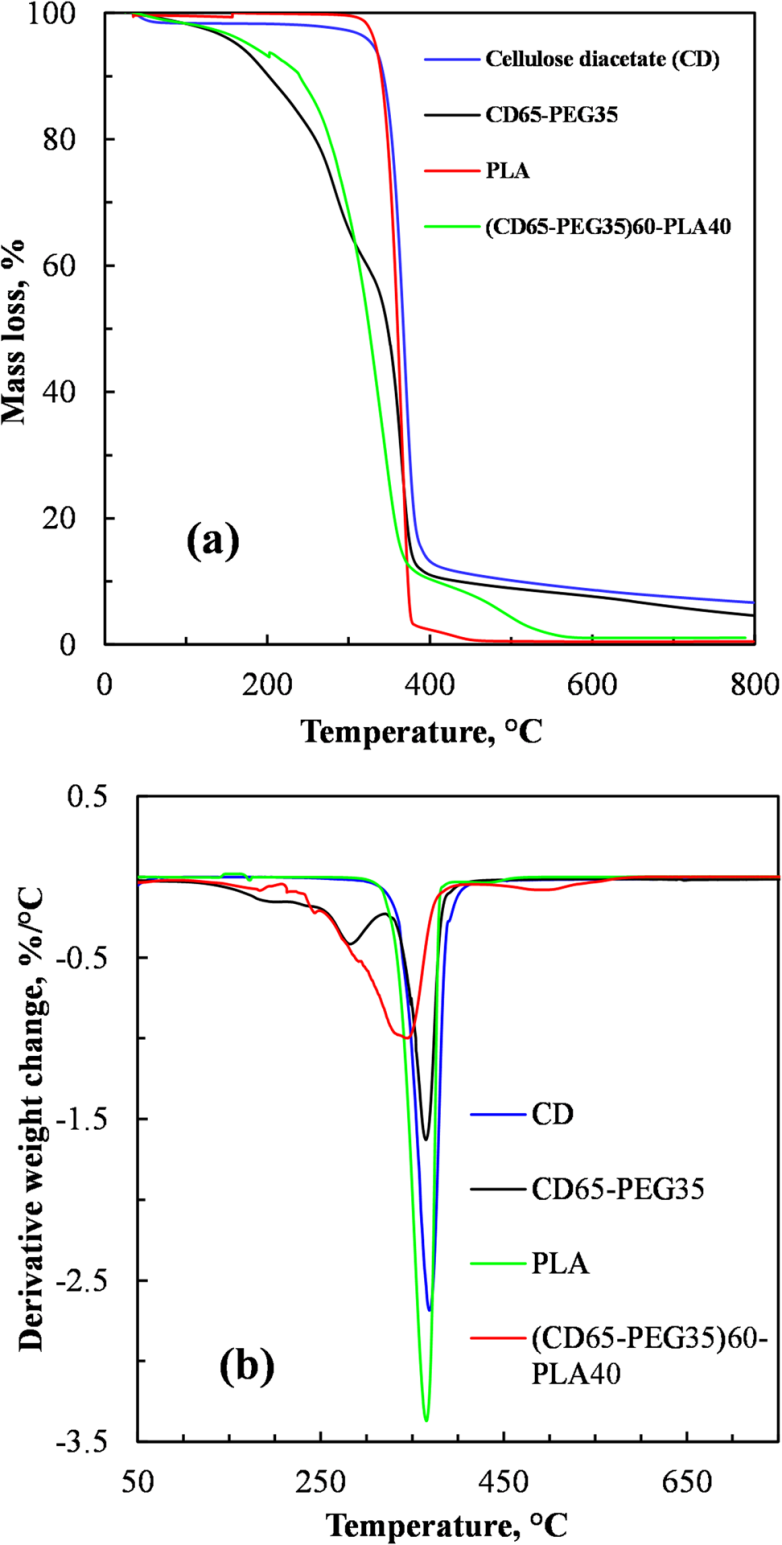
**a** TGA and **b** derivative TG of CD, PEG plasticized CD (CD65-PEG35), PLA and PEG plasticized CD blended with PLA at 40 wt% ((CD65-PEG35)60-PLA40)

Figures 7a and b shows that that the onset temperature for thermal degradation for the PEG plasticized CD blended with PLA at 40 wt% ((CD65-PEG35)60-PLA40) was around 120 °C, similar to the onset temperature for degradation of the PEG plasticized CD.

However, the onset temperature for degradation of the PEG plasticized CD blended with PLA at 40 wt% was lower by almost 280 °C, compared to that of the neat CD and the neat PLA. These observations indicated that blending the PEG plasticized CD with PLA at 40 wt% deteriorated the thermal stability of the PLA. These could be attributed to the presence of more PEG plasticizer in the blend (CD65-PEG35)60-PLA40, compared to the blend (CD65-PEG35)40-PBAT60). Therefore, blending the PEG plasticized CD with PLA at 40 wt% did not improve thermal stability.

### X-Ray Diffraction

XRD was used to study the structural properties of the neat CD, PEG plasticised CD, PBAT, PLA and the PEG plasticized CD blends with PLA and PBAT. Cellulose acetates with low DS are known to be amorphous, whereas highly substituted cellulose acetates such as CTA with DS of 3 are semi-crystalline [19]. Crystallinity of cellulose acetates increases with the DS [19]. X-ray diffractograms of CD with a DS of 2.45 (CD) exhibit three crystalline peaks with *d* spacings 6.7, 8.4 and 10.4 Å which are identified using the literature data of the crystalline phase of CTA as a reference (Fawcett et al. 2013). These peaks are characterized by weak intensities and are imbedded within humps in the x-ray diffraction patterns of CD [19]. This shows that CD has a low degree of crystallinity. Bao [19] reported a degree of crystallinity (DC) of 9–16% for cellulose acetate (CD) with a DS of 2.45.

**Fig. 8:**
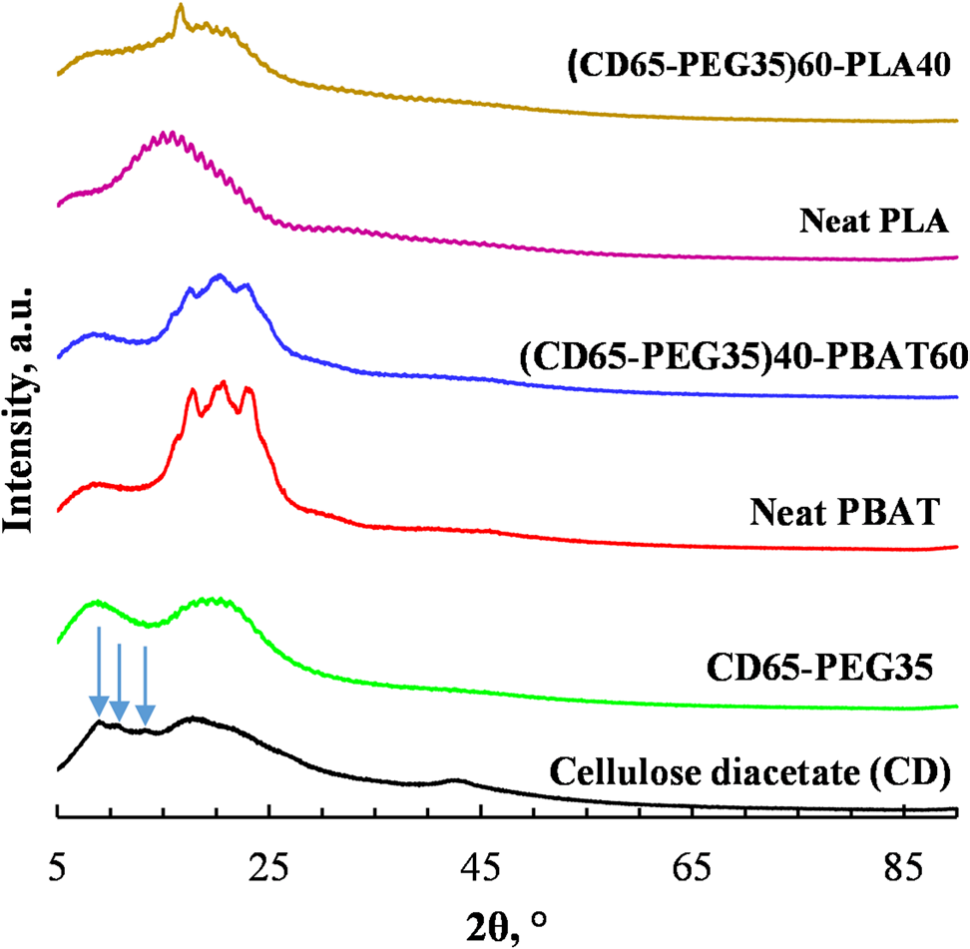
XRD patterns of CD and PEG plasticized CD (CD65-PEG35) (black arrows show the three characteristic cellulose crystalline peaks), PBAT and PEG plasticized CD blended with PBAT at 60 wt% ((CD65-PEG35)40-PBAT60), and PLA and PEG plasticized CD blended with PLA at 40 wt% ((CD65-PEG35)60-PLA40)

The XRD pattern of the CD used the present study is shown in Fig. 8. It exhibits the three peaks that are expected in highly substituted cellulose acetates at 2θ diffraction angles of about 8°, 10° and 12° as indicated in Fig. 8. The intensities of the peaks are very weak, showing that the CD used in the present study had low crystallinity. The three characteristic humps of amorphous cellulose derivative present in the XRD pattern of the CD in Fig. 8 show that it was highly amorphous. Hence the CD used in the present study was a semi-crystalline polymer, which was highly amorphous [19, 37, 45].

Plasticization weakens the intermolecular interactions between the polymer molecular chains and disrupts their ordering, which is a prerequisite for polymer crystallinity. Hence the three crystallization peaks initially observed in the CD were not observed in the XRD patterns of the plasticized CD.

Figure 8 also shows the XRD patterns of the two selected blends, compared to those of the neat polymers CD, PBAT and PLA. PBAT exhibits four characteristic diffraction peaks in its XRD pattern between ca. 2θ 17–25°, which are due to its crystalline structure [46]. The PBAT utilized in this study whose XRD pattern is presented in Fig. 8 showed three diffraction peaks in its XRD pattern, the fourth one was not apparent, although it appeared as a weak shoulder. The intensities of these peaks were much lower in the XRD pattern of the PEG plasticized CD blended with PBAT at 60 wt% ((CD65-PEG35)40-PBAT60), indicating that the crystallinity of the blend was relatively lower compared to that of PBAT. It was established earlier on that the CD used in the present study had low crystallinity, and incorporating the plasticizers had the effect of reducing this crystallinity, making the material more amorphous.

In Fig. 8, the characteristic crystalline diffraction peak of PLA was observed at 2θ 15°. This characteristic peak was broad, indicating that crystalline domains were embedded within a highly amorphous structure in the PLA. The PLA characteristic peak was also observed in the PEG plasticized CD blended with PLA at 40 wt%, flanked by broad shoulders which were earlier identified as broad peaks in the XRD pattern of the neat CD and the PEG plasticized CD. Based on this analysis, the PEG plasticized CD blended with PLA at 40 wt%, was semi-crystalline, although it was highly amorphous.

### Fourier-Transform Infrared Spectroscopy

**Fig. 9:**
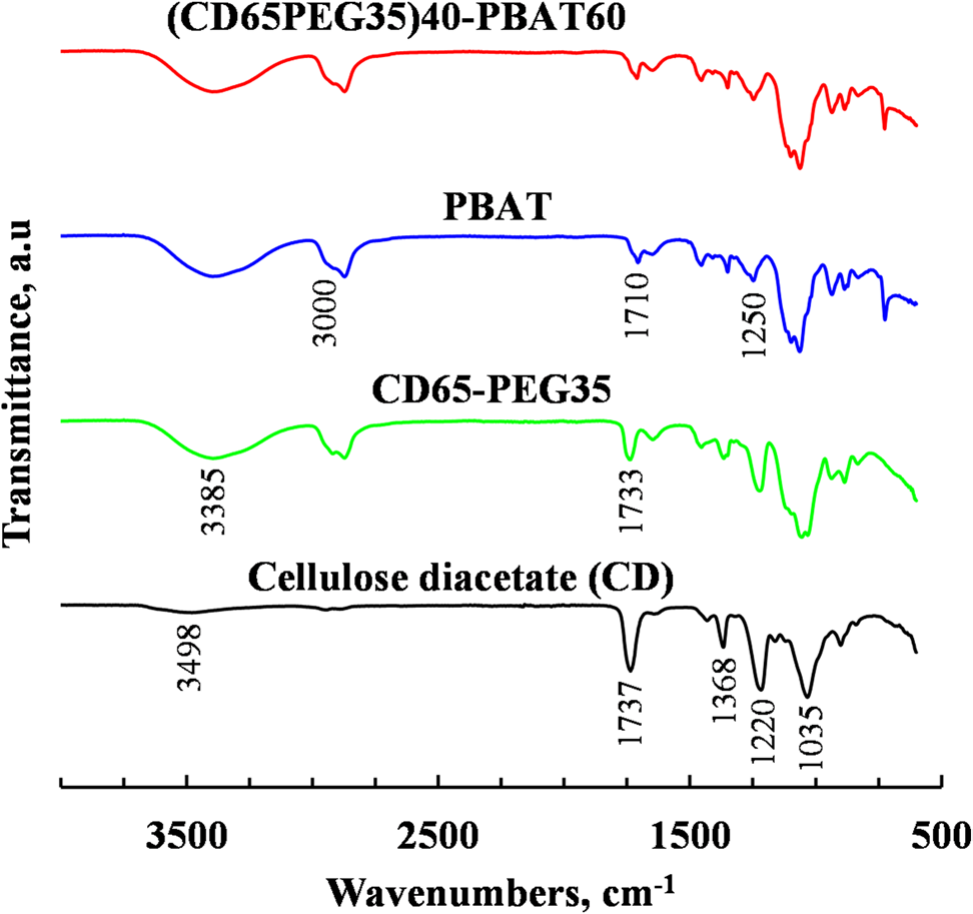
FT-IR spectra of CD, CD plasticized with 35 wt% PEG (CD65-PEG35), PBAT and CD plasticized with 35 wt% PEG blended with PBAT at 60 wt%

The FT-IR spectra of the neat CD and its PEG plasticized form are presented in Fig. 9. The three main acetyl group vibrations (C=O, C–H and C–O bond stretching) were observed at wavenumbers 1737, 1368 and 1220 cm^−1^ respectively. The peaks observed at 3489 cm^−1^ and 1035 cm^−1^ were attributed to the O–H bond stretching of unacetylated hydroxyl groups and the C–O–C ether linkage in-between the AGU units of CD, respectively [23].

Figure 9 further showed characteristic peaks for hydroxyl and carbonyl groups that are found in PEG at wavenumbers of 3385 and 1733 cm^−1^ respectively, in the PEG plasticized CD. There was a shift in the CD characteristic peak (hydroxyl group) to the lower wavenumbers, in the PEG plasticized CD. This shift and the peak broadness were attributed to hydrogen bonding interactions between the oxygens in acetyl groups in the CD and the OH-end groups of PEG [23, 47].

The FT-IR spectra of the neat PBAT shown in Fig. 9 showed the characteristic PBAT peaks. A peak was observed at around 3000 cm^−1^ wavenumbers, representing the C–H stretching of both aliphatic and aromatic components in PBAT. The carbonyl groups were observed at wavenumbers 1710 cm^−1^ and C–O stretching was observed at wavenumbers at 1250 cm^−1^, representing ester linkage, respectively.

In polyesters such as PBAT, hydrolysis of ester-linkages is the dominant degradation mechanism between 150 and 215 °C followed by main-chain scission at temperatures above 180 °C [48]. The PBAT and the blends synthesized in this study were processed at temperatures lower than 200 °C. TGA of the PBAT used in this study did not show any evidence of the degradation mechanisms mentioned above. The FT-IR spectra in Fig. 9 showed no significant bond formation or disappearance, which is further evidence that degradation did not occur during processing.

**Fig. 10:**
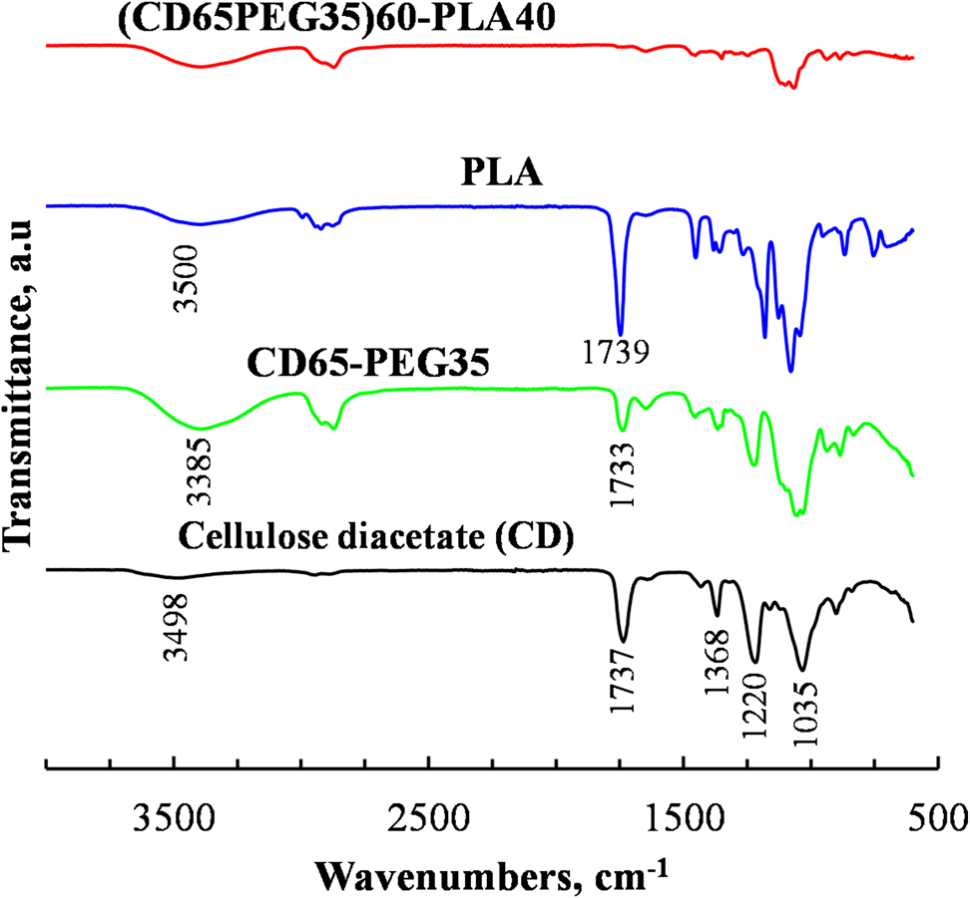
FT-IR spectra of CD, CD plasticized with 35 wt% PEG (CD65-PEG35), PLA and CD plasticized with 35 wt% PEG blended with PLA at 40 wt%

Characteristic infrared absorption peaks of PLA were observed in the FT-IR spectra of PLA presented Fig. 10. These peaks were observed at wavenumbers 3500 cm^−1^ due to O–H stretching (small terminal hydroxyl groups in the main PLA backbone chain) and at 1739 cm^−1^ due to C=O vibrations, similar to the observations by Wisam et al. (2010). The carbonyl groups and C-O bonds observed at wavenumbers 1750 cm^−1^ and 1220 cm^−1^ in the FT-TR spectra of CD were not present in the FT-IR spectra of PEG plasticized CD blended with PLA at a PLA content of 40 wt%. This could be attributed to deacetylation of CD during injection moulding or partial degradation of plasticized CD. The disappearance of the carbonyl peaks in the FT-IR spectra of plasticized CD is regarded as a typical degradation path of CD [49–51].

### Scanning Electron Microscopy

**Fig.11:**
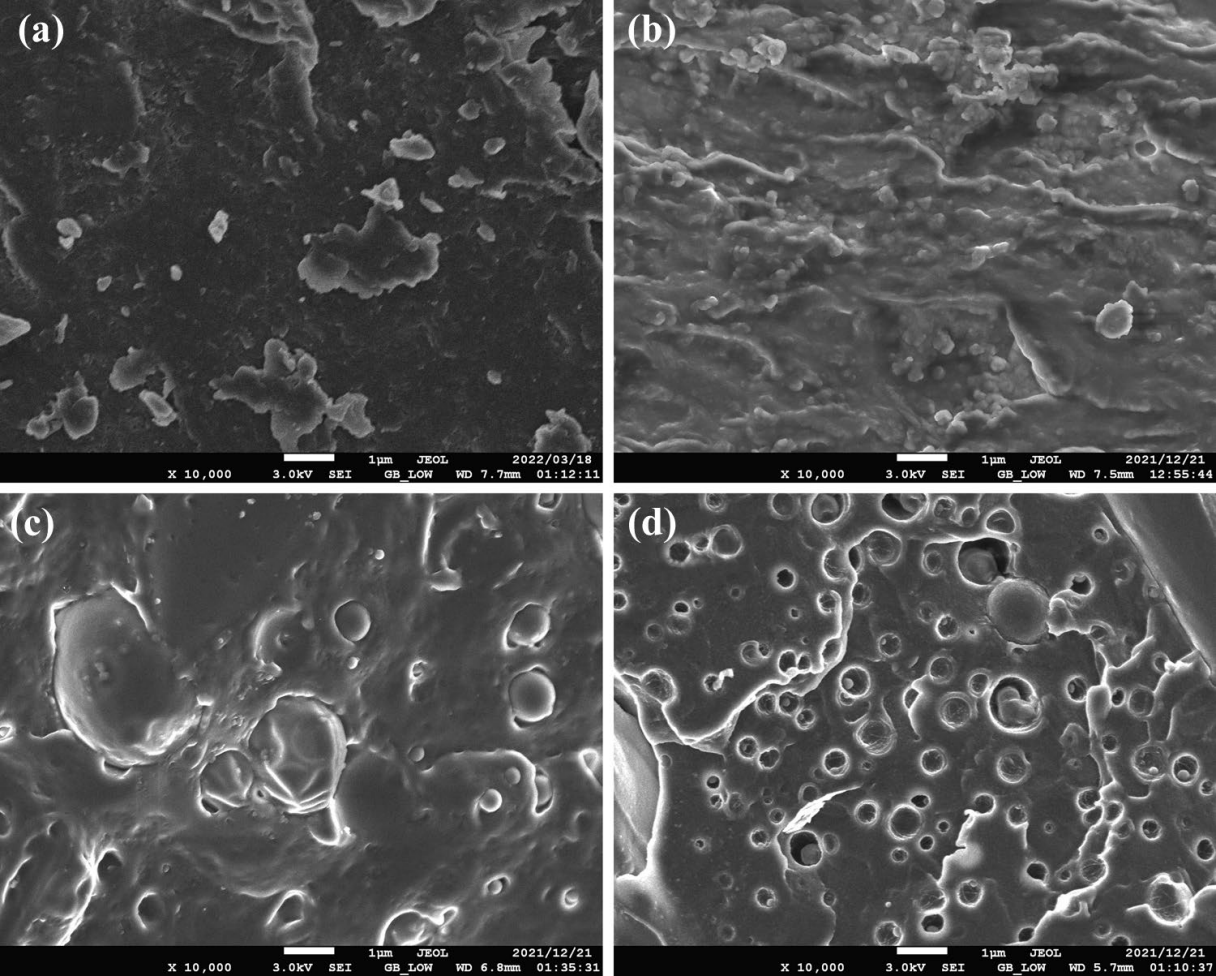
Scanning electron micrographs of neat **a** CD, **b** CD plasticized with 35 wt% PEG (CD65-PEG35), **c** CD plasticized with 35 wt% PEG blended with PBAT at 60 wt% PBAT ((CD65-PEG35)40-PBAT60) and **d** CD plasticized with 35 wt% PEG blended with PLA at 40 wt% PLA ((CD65-PEG35)60-PLA40)

Figure 11 shows the scanning electron micrographs of neat CD, PEG plasticized CD, PEG plasticized CD blended with PBAT at 60 wt% and PEG plasticized CD blended with PLA at 40 wt%. Figure 11b shows that, compared to the neat CD in Fig. 11a, the PEG plasticized CD in Fig. 11b apparently exhibited some particulates within a homogeneous matrix. These particulates could have been unplasticized CD. However, there was no discernible phase separation in Fig. 11 (b), showing that the PEG plasticizer was compatible with CD.

In Fig. 11c, which was the micrograph of the PEG plasticized CD blended with PBAT at 60 wt%, relatively large spherical particles imbedded in a homogenous matrix were observed. The micrograph in Fig. 11c showed an apparent good interfacial interaction between the spherical particles and the homogenous matrix. This could be regarded as evidence of compatibility and partial miscibility of the PEG plasticized CD with PBAT at 40 wt%.

Figure 11d, the micrograph of PEG plasticized CD blended with PLA at 40 wt%, showed that the blend was composed of relatively small spherical particles embedded in a continuous matrix. However, there was clear evidence that some of the spherical particles fell out when the blend was fractured, leaving spherical voids with the polymer matrix. Figure 11d shows that there was poor interfacial interaction between the spherical particles and the polymer matrix. This is evidence that there was poor compatibility between the PEG plasticized CD blended with PLA at 40 wt%.

### Biodegradation Studies of CD and its Blends

#### Disintegration of Film Samples

The primary step in the biodegradation of a plastic material is its disintegration into smaller fragments. Thus, a disintegration test is used to evaluate if a plastic material undergoes this primary biodegradation step, whether it breaks into small pieces or not, with lower molecular weights which are then easily attacked by microorganisms in the subsequent biodegradation steps.

**Fig. 12:**
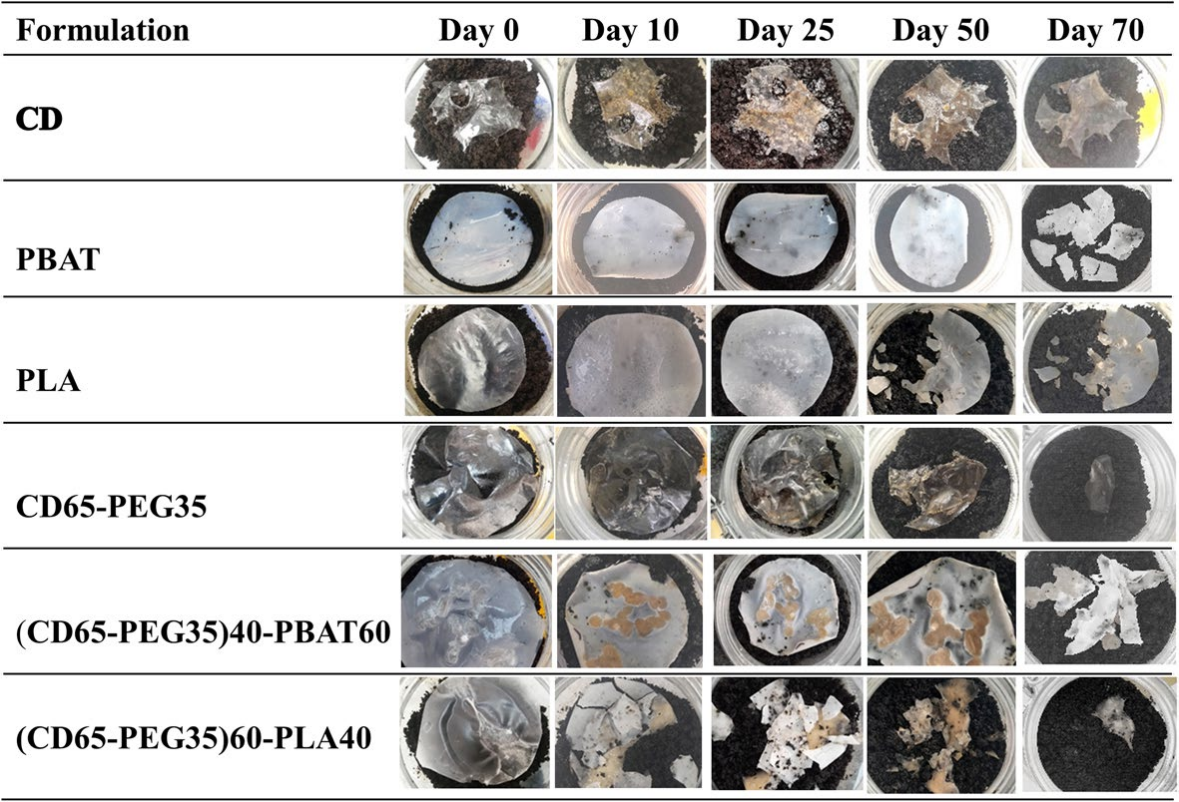
Progression of the disintegration of CD and its blends under simulated aerobic composting

In the present study, disintegration tests were done on film samples of neat CD, PEG plasticized CD, neat PLA, neat PBAT, PEG plasticized CD blended with PBAT at 60 wt% and PEG plasticized CD blended with PLA at 40 wt%. Figure 12 shows the progression of the disintegration of the materials described above over an incubation period of 70 days.

The occurrence of a white surface on the compost in the composting reactor with CD and the high tightening of its lid was evidence of an elevated evolution of CO_2_. However, no visible cracks or pieces were observed in the CD film over the 70-day incubation period as seen in Fig. 12.

The PBAT film began to be fragile and started breaking up at day 70 as shown in Fig. 12. The PLA film started to show cracks after day 25, became fragile and started breaking up beyond day 50. The disintegration behaviour of PBAT and PLA corresponded to their degradation rates, as discussed later on.

It was observed that PEG plasticized CD became thinner but not fragile beyond day 50. This disintegration behaviour corresponded with its biodegradation rate discussed later on, which was initially slow and only started to pick up beyond day 60. The PEG plasticized CD blended with PBAT at 60 wt% and PEG plasticized CD blended with PLA at 40 wt% were partially soluble in the chloroform solvent. The PEG plasticized CD blended with PBAT at 60 wt% became fragile at day 70 (Fig. 12), whereas the PEG plasticized CD blended with PLA at 40 wt% started cracking and breaking up at day 10 and became fragile beyond day 50 (Fig. 12). This behaviour corresponded well with the disintegration behaviour of neat PBAT, and PLA as seen in Fig. 12. The disintegration behaviour of the two blends was also in agreement with the biodegradation rates observed and discussed in the subsequent sections.

#### CO_2_ Evolution

**Fig. 13:**
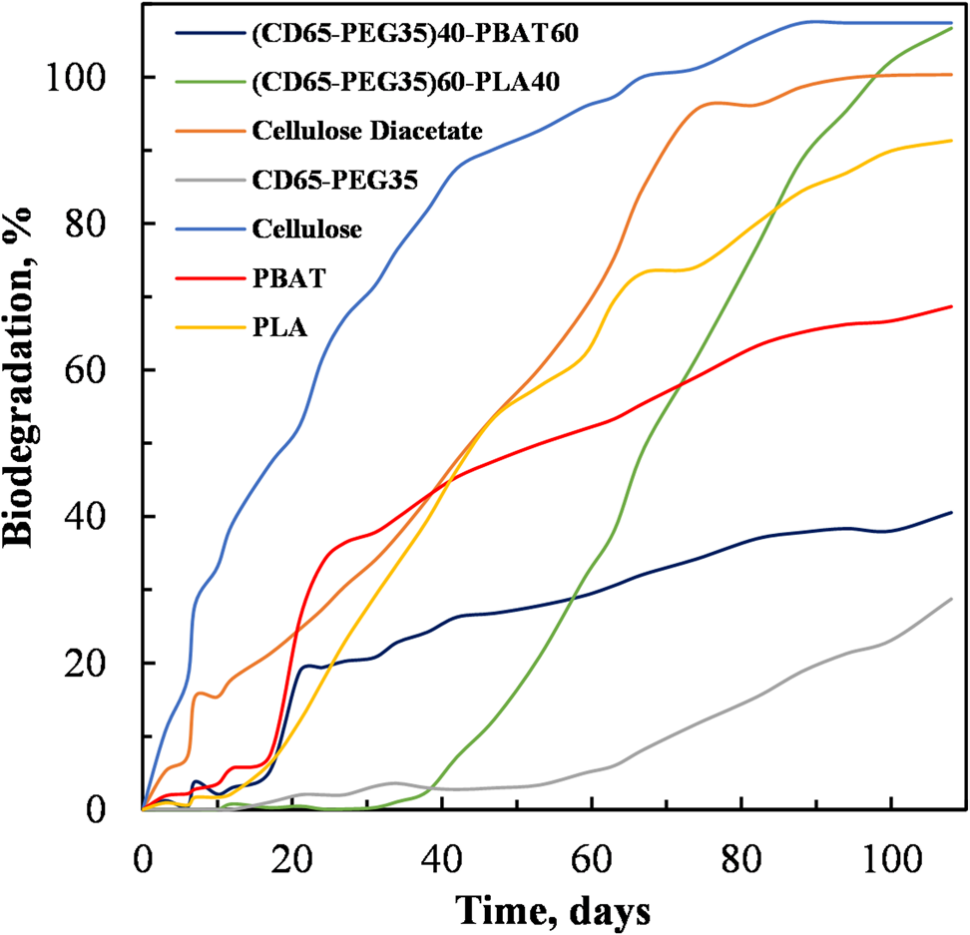
Biodegradation curves for MCC, CD, PBAT, PLA, PEG plasticized CD (CD65-PEG35), PEG plasticized CD blended with PBAT at 60 wt% PBAT ((CD65-PEG35)40-PBAT60) and PEG plasticized CD blended with PLA at 40 wt% PLA ((CD65-PEG35)60-PLA40) under simulated aerobic composting

Figure 13 and Table 5 show the percentage of biodegradation of the test samples during the incubation period of 108 days as measured by a titration method under controlled composting conditions. The biodegradation results showed that there was no appreciable CO_2_ emission, implying no biodegradation, in all test samples excluding the cellulose (positive reference material) and CD for the first 15 days. The results show that an incubation (lag) phase took place, wherein the microorganisms acclimatised to conditions for the first 15 days.

**Table 5:** Test materials composition and their biodegradability

Sample Name	Carbon (g)	Carbon (%)	Th CO_2_ (g)	Biodegradation (%)
MCC	7.510	45.902	12.7	107.41
CD	7.5034	51.421	14.1	100.32
CD65-PEG35	7.5054	52.326	14.4	28.75
PLA	7.5097	55.034	15.2	91.33
PBAT	7.5009	68.638	18.9	68.65
(CD65-PEG35)60-PLA40	7.4971	53.888	14.8	106.66
(CD65-PEG35)40-PBAT60	7.5171	61.552	17.0	40.56

For (CD65-PEG35)60-PLA40 and CD65-PEG35, no significant degradation was observed for the first 40 and 65 days of the incubation period respectively, with biodegradation reaching below 5%. In contrast, (CD65-PEG35)40-PBAT showed a fairly higher degradation rate, reaching 30% in the first 50 days of incubation. CD, PLA and PBAT showed higher rates of degradation reaching 50–90% biodegradation when compared to other samples during the first 70 days of the incubation period. However, it is noticeable that (CD65-PEG35)60-PLA40 showed a high biodegradation rate (more than the rest of the test samples) after a prolonged lag phase. The obtained results clearly indicate that for the first 70 days the primary degradation process was mainly due to the materials composition, in which the long polymeric molecules broke into low molecular weight compounds, including oligomers, trimers, dimers, and monomers.

The degradation of acylated cellulose polymers, such as CD, is influenced by their DS, which reduces the rate of biodegradation but does not inhibit biodegradation of CD [52]. Whereas hetero-chain polymers such as PLA undergo random non-enzymatic polymer chain scission and hydrolysis of ester groups which results in molecular weight reduction, leading to more enzymatic activities (high biodegradation) [53]. PBAT is known to have a long lag phase, up to 20 days with its acceleration stage starting after 60 days and only reaching 92.0% biodegradation after a 200 days incubation period [54]. Furthermore, during the hydrolytic biodegradation mechanism, PBAT monomer components are reported to degrade under different rates [55].

Interestingly, the cellulose positive reference materials showed more than 70% biodegradation within 45 days. This indicates that the biodegradation set-up adopted meets the validity of test method requirements [33]. Finally, the exponential degradation phase started after 70 days for all the tested samples, where it shows CD, CD65-PEG, PLA, PBAT, (CD65-PEG35)60-PLA40 and (CD65-PEG)40-PBAT60 reached 100.32, 28.75, 91, 68.65, 106 and 40% biodegradation, respectively (Table 5). During this exponential phase (secondary step or mineralization), the low molecular weight compounds were being assimilated into microbes, yielding CO_2_. In addition, the rate of biodegradation of each of the test samples mainly depended on the polymer sample compositions of crystalline and amorphous regions.

PEG is a water-soluble hydrophilic plasticizer with polar OH groups. The hydrophilic nature of the PEG had the potential to increase the permeability of water and oxygen into the plasticized CD, thereby accelerating its disintegration and biodegradation rates, compared to the neat CD [56]. However, it was noted in this study that the PEG plasticized CD (CD65-PEG35) had a slower biodegradation rate compared to the neat CD, in contrast to several studies in which PEG was used as a plasticizer and was observed to accelerate the biodegradation rates [30, 57, 58]. The water permeability of PEG depends on its molecular weight. Relatively lower permeabilities have been reported for low molecular weight PEG (PEG 300) in comparison to high molecular weight PEGs [56]. The PEG used in this study had a molecular of 200 g/mol, which was relatively low. Low molecular weight PEGs such as the one used in this study could possibly bond well with the CD through hydrogen bonding, limiting their water permeability capacity, thereby by retarding the biodegradation rate of the plasticized CD [56]. The FT-IR results of the CD and CD65-PEG35 in Fig. 9 suggest that hydrogen bonding was present in the PEG plasticized CD. SEM results in Fig. 11 also show evidence of good interfacial interaction between the CD and PEG. High molecular weight PEGs are not able to position themselves well in-between the polymer segments to sufficiently form hydrogen bonds, thus they are available to increase the permeability of water and oxygen, thereby accelerating disintegration and biodegradation rates [56, 59].

The biodegradation rate of the (CD65-PEG35)60-PLA40 blend was higher compared to that of the PLA (Table 5) suggesting that blending the CD65-PEG35 with PLA accelerated the biodegradation rate of PLA. The XRD results in Fig. 8 showed that blending the PEG plasticized CD (CD65-PEG35) with the PLA suppressed the crystallinity of the PLA. It is known that the biodegradation of polymers occurs faster in the amorphous phases [56, 60]. The amorphous phases of polymers absorb fluids more easily than the crystalline phases, hence degrade faster (low energy required). Also, the uniformly packed molecules associated with the crystalline phases define the degradation mechanism.

The biodegradation rate of the (CD65-PEG35)40-PBAT60 blend was lower than that of the neat PBAT (Table 5). Figure 8 shows that the (CD65-PEG35)40-PBAT60 blend retained an XRD pattern similar to that of the PBAT, probably due to the blend having a high PBAT content. The XRD pattern of the blend showed that its degree of crystallinity was not much different from that of the PBAT, which could have contributed to is lower biodegradation rate [56, 60]. Also the SEM micrograph of the blend in Fig. 11c suggests that there was good interfacial interaction between the components, which could possibly have limited water permeability capacity, thereby retarding the biodegradation rate of the blend [56].

## Conclusions

This study has shown that melt processing of CD in the form of extrusion and injection moulding can be easily conducted by plasticizing it using PEG-200. The PEG significantly reduced the glass transition temperature of CD from ca. 220 °C to less than 100 °C, thereby improving its melt processibility. However, plasticizing CD with PEG reduced its thermal stability and diminished its crystallinity. Furthermore, PEG plasticized CD was found to be relatively ductile.

The present study also showed that blends of PEG plasticized CD at 35 wt% PEG with PBAT and PLA can be easily melt processed over the 0–100% composition range. The tensile strength of PEG plasticized CD blended with PLA increased with an increase in the PLA content. This suggested that the PEG plasticized CD was partially miscible and PLA. The tensile strength of PEG plasticized CD blended with PBAT decreased with an increase in the PBAT content, exhibiting a minimum at 60 wt% PBAT, suggesting that PEG plasticized CD was immiscible with CD. This was however in contrast with the SEM observations, which showed suggested good interfacial interaction in the blend. Blending PEG plasticized CD with PBAT at 60 wt% slightly improved its thermal stability. However, blending PEG plasticized CD with PLA at 40 wt% slightly diminished its thermal stability.

Disintegration tests conducted on film samples of the CD and its blends agreed with the biodegradation tests as manifested by evolution of CO_2_ during the simulated aerobic composting. According to the ASTM D5338 standard, the CD and PLA used in this study were biodegradable materials. The PBAT used in this study exhibited a slower biodegradation rate over a 108-day incubation period, undergoing a degradation of about 70%. Plasticization of CD with PEG suppressed its biodegradation as PEG plasticized CD did not undergo any appreciable biodegradation for close to 60 days, its biodegradation rate only started to rise after the 60-day incubation period, reaching only 28.75% after an incubation period of 108 days. If it were to continue biodegrading at that rate, it is unlikely that it would meet the ASTM D5888 biodegradability.

The PEG plasticized CD blend with PBAT at 60 wt% exhibited an almost 20-day phase lag in its biodegradation and a subsequent relatively slower biodegradation rate, which was however higher than that of the PEG plasticized CD. The biodegradation rate of this blend after the 108-day incubation period shows that it was likely to have biodegraded to over 90% after the 180-day period required for it to meet the ASTM D5888 standard for biodegradability. It can therefore be confidently concluded that it is a biodegradable plastic. The PEG plasticized CD blend with PLA at 40 wt% initially exhibited a prolonged lag phase of about 40 days, however, its biodegradation rate accelerated after this phase to more than 100%, within the 108-day incubation period. This blend was biodegradable as per the ASTM D5888 standard. It can thus be concluded that melt processible, biodegradable CD blends can be synthesized through plasticization with PEG and blending with PBAT and PLA.

## Data Availability

The datasets generated during and/or analysed during the current study are available from the corresponding author on reasonable request.
